# Application of Improved Wavelet Thresholding Method and an RBF Network in the Error Compensating of an MEMS Gyroscope

**DOI:** 10.3390/mi10090608

**Published:** 2019-09-13

**Authors:** Guangrun Sheng, Guowei Gao, Boyuan Zhang

**Affiliations:** 1 Beijing Key Laboratory of Sensors, Beijing Information Science & Technology University, Beijing 100101, China; shengguangrun@163.com (G.S.); zbybvn@163.com (B.Z.); 2 Key Laboratory of Modern Measurement & Control Technology, Ministry of Education, Beijing Information Science & Technology University, Beijing 100192, China

**Keywords:** MEMS gyroscope, wavelet threshold de-noising, RBF neural network, inertial navigation system

## Abstract

The large random errors in Micro-Electro-Mechanical System (MEMS) gyros are one of the major factors that affect the precision of inertial navigation systems. Based on the indoor inertial navigation system, an improved wavelet threshold de-noising method was proposed and combined with a gradient radial basis function (RBF) neural network to better compensate errors. We analyzed the random errors in an MEMS gyroscope by using Allan variance, and introduced the traditional wavelet threshold methods. Then, we improved the methods and proposed a new threshold function. The new method can be used more effectively to detach white noise and drift error in the error model. Finally, the drift data was modeled and analyzed in combination with the RBF neural network. Experimental results indicate that the method is effective, and this is of great significance for improving the accuracy of indoor inertial navigation based on MEMS gyroscopes.

## 1. Introduction

Since the 1970s, when the concept of MEMS was put forward, MEMS gyroscopes have been widely used in military fields and civil fields [1]. Because they are small in size, low in cost, and simple in structure [2], in inertial navigation technology, the gyro is one of the core pieces of equipment for modern precision navigation, guidance, and control systems, especially in the application of indoor navigation [3]. However, the limited structural defects and processing technology cause a large random error in the gyroscope [4]. When the system is working for a long time, the random error of the gyroscope will accumulate over time, resulting in errors or even failures of the indoor inertial navigation system. An effective and feasible method is to use the filtering technique to compensate the random error in real time. Therefore, the accurate modeling and compensation of a gyroscope’s random errors becomes an effective method to achieve higher navigation accuracy based on MEMS device accuracy [5]. This is great for improving the accuracy of indoor inertial navigation systems.

Gyro drift has the characteristics of random time-varying, so it is necessary to analyze the error and noise characteristics of the gyroscope. The Allan variance method is a time domain analysis technique with obvious advantages in analyzing random errors. In recent years, scholars have used Allan variance to analyze the error of inertial devices [6]. Then, the wavelet threshold is used to separate the white noise from the drift error. The hard and soft threshold denoising method was proposed by David L. Donoho and Iain M. Johnstone, but the threshold denoising method was not improved [7]. Some scholars have proposed improved methods, such as the semi-soft threshold function [8], Xing threshold function [9], and genetic adaptive threshold function [10]; however, these threshold functions are simple to handle on different scales and have poor adaptability. Therefore, it is necessary to propose an improved threshold denoising method that can be applied to an indoor inertial navigation system, which can improve system navigation accuracy. The radial basis function neural network (RBF) is also applicable to the stochastic process of MEMS gyroscopes because of its nonlinear, adaptive, and self-learning characteristics; if the improved wavelet threshold denoising method is compared with the RBF neural network, it can effectively compensate the random drift error, which is of great significance for improving the accuracy of the indoor inertial navigation system based on MEMS gyroscopes.

This paper is devoted to the research of inertial signals. Compared with the traditional method, the method improves the utilization of high-frequency effective signals and has better adaptability. The paper is organized as follows. Firstly, the Allan analysis of variance is used to perform random error analysis on the signal output from a MEMS gyro. Then, the advantages and disadvantages of conventional wavelet threshold denoising are analyzed and an improved method is proposed, separating white noise and gyroscope drift errors with improved threshold functions. The gradient error model is then modeled by using a gradient radial basis (RBF) neural network. The RBF neural network has strong nonlinear processing capability [11], which allows for more effective error compensation for MEMS gyros. Finally, the feasibility and effectiveness of the improved method are proven by experiments and simulation analysis.

## 2. MEMS Gyroscopes Allan Variance Analysis

The Allan variance method was originally used to study the phase and frequency instability of the oscillator [12]. Allan variance becomes a measure of sensor output stability if inertial sensors are configured to vibrate at their resonance (frequency output sensors), unless there is not any use of Allan variance in amplitude modulation [13]. Therefore, Allan variance is widely used in noise analysis and performance evaluation of MEMS inertial sensors [14]. In this paper, a simple Allan square difference segment fitting method was used to estimate the noise figure of the gyroscope. This method can avoid a lot of calculations, can intuitively read out various noise coefficients, and effectively avoid calculation errors.

(1) The output angular rate of the MEMS gyro is collected at the sampling interval $T_{s}$ to obtain a sample space with a total length of T, and the number of sampling points is N = T/$T_{s}$;

(2) Each successive n (n = 1, 2, 3..., N, N < T/2) data are used as a subset to average the sample space data, a total of J = rounded (T / n) subsets can be divided. Each set of data is averaged to obtain a set of random variables whose elements are group averages:

(1) $${\bar{\omega }}_{k}(n)\;=\;\frac{1}{n}\sum_{i=1}^{n}\omega_{\left(k-1\right)n+i\;},\;k\;=\;1,\;2,\;\ldots ,\;j$$

(3) The Allan variance is calculated for each different averaging time:

(2) $$\sigma^{2}\left(\tau_{m}\right)\;=\;\frac{1}{2\left(k-1\right)}\sum_{k=1}^{k-1}{\left[{\bar{\omega }}_{k+1}\left(n\right)-{\bar{\omega }}_{k}\left(n\right)\right]}^{2}$$

In the formula, K is the number of divided subsets. The duration of each data subset is expressed as $\tau_{m}$ = n$T_{s}$ [15].

**Figure 1:**
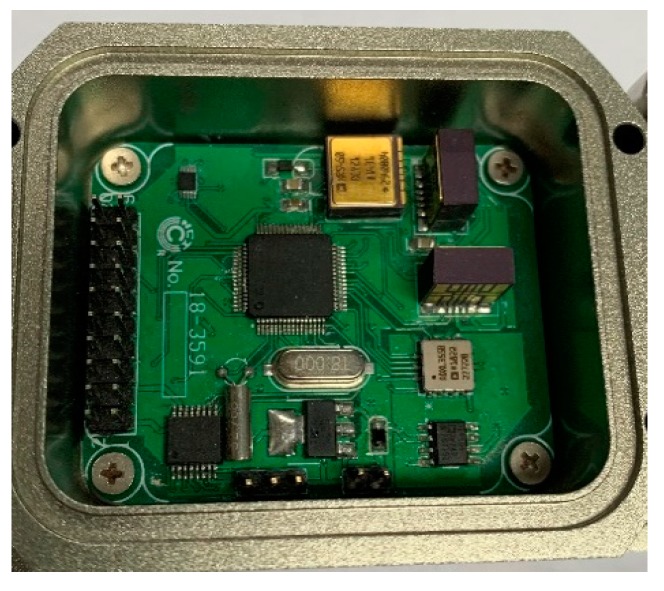
Inertial measurement sensor.

**Figure 2:**
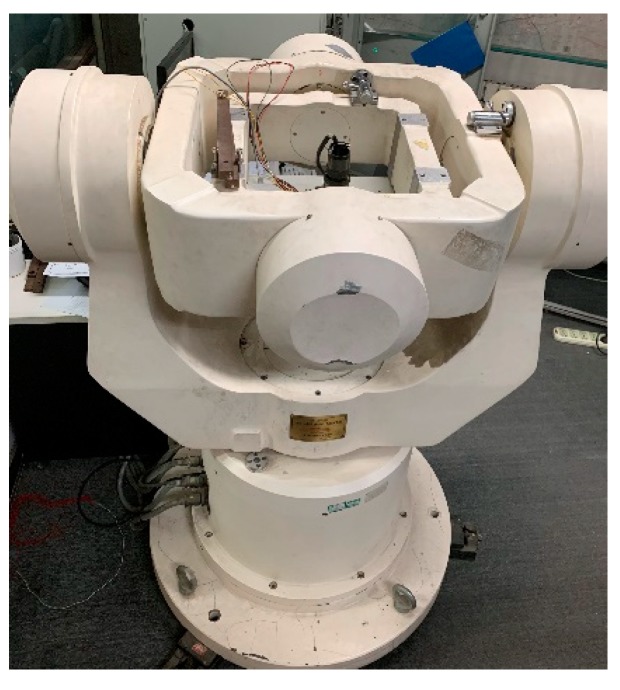
Setup equipment used during the test.

In this paper, an inertial measurement sensor, using a combination of an ADXRS450E gyroscope and an ADXL354 accelerometer manufactured by Analog Devices, with the three MEMS gyroscopes in orthogonal relationship, was used to measure the carrier angular velocity. The MEMS inertial sensor was placed statically on the three-axis turntable, the data acquisition frequency set to 50 Hz, the gyroscope data preheated for 1 h. There was continuous acquisition of the gyroscope data for three hours, and the above process was repeated for five days. A total of 30 minutes of data were taken as a sample and the Allan analysis of variance was performed. Figure 1 and Figure 2 show the measurement sensors and experimental equipment used during the test.

**Figure 3:**
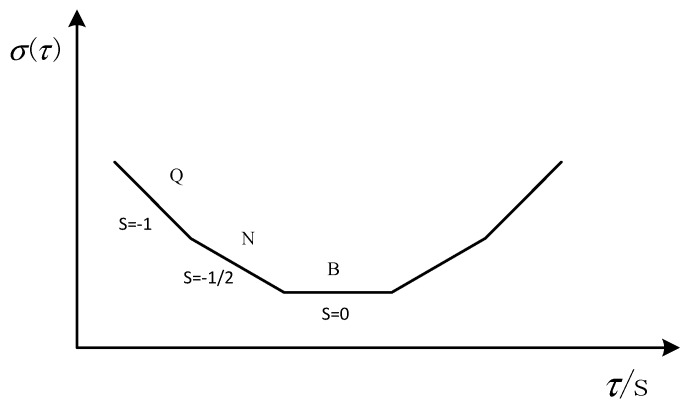
Diagram of the Allan mean square deviation for the MEMS gyroscope random error.

Allan’s mean square logarithm plot can clearly depict the various error components of the gyroscope, there were different error terms and different time intervals, and the slope of the double logarithmic curve was different. In Figure 3, S is the slope of the curve. The slope of −1 corresponds to the gyro quantization noise. The part with a slope of −1/2 corresponds to the angle random walk, the part with a slope of 0 corresponds to the zero offset instability [16]. They are represented by Q, N, and B, respectively.

**Figure 4:**
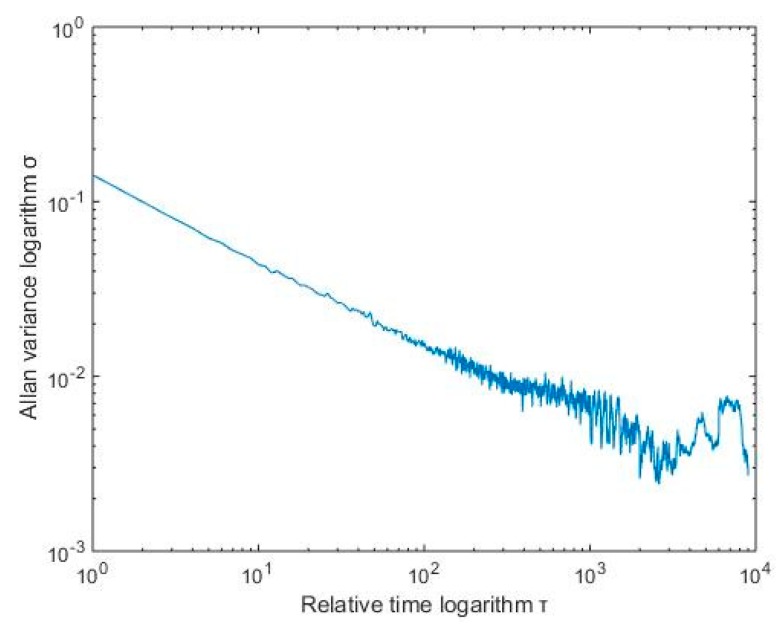
X-axis Allan analysis of variance of the MEMS gyroscope.

Figure 4 shows the Allan standard deviation double logarithmic curve of the X-axis gyro, Taking the quantization noise as an example, the specific calculation method was as follows: According to Figure 3, Q corresponds to the portion of the Allan mean square error logarithm curve with a slope of −1. After the straight line fitting of the quantization noise was completed, the variance formula according to the quantization noise was $\sigma_{N}^{2}\left(\tau \right)=\frac{N^{2}}{\tau }$, which took an average time of $\sqrt{3}$ s, then found Q. After the above processing steps, different error term coefficients of various MEMS gyroscopes were obtained. The results are shown in Table 1.

## 3. Improved Wavelet Threshold Denoising Method

### 3.1. Wavelet Threshold Denoising

In the actual computer control system, the sampling signal is polluted by various noises and interferences. Getting a “pure” signal from these noise-interfering signals is the key to establishing a high-precision model of the system and achieving high-performance control.

The wavelet threshold denoising method is based on the fact that the wavelet coefficients of signals and noise at a certain scale have different characteristics, and the signal containing noise is wavelet transformed on a certain scale. After the transformation, the real signal generally exists in a large value and a small number of low-frequency wavelets. Among the coefficients, the noise signal generally exists in the high-frequency coefficient, with a small amplitude and a large number. Wavelet threshold denoising is to set the threshold on different scales of wavelet decomposition [17]. It is considered that a wavelet coefficient smaller than the threshold is a noise signal, which can be set to 0. If it is greater than the set threshold, it belongs to the real signal, and directly preserves or performs compression transformation. Then, the processed wavelet coefficients are inversely transformed by the wavelet to obtain a filtered signal.

The wavelet threshold denoising method generally includes the following three steps:

(a) Wavelet decomposition with noise signal, select an appropriate wavelet base, determine the level N of wavelet decomposition, process the signal, and obtain the wavelet coefficient;
(b) Quantify the high frequency coefficients of the wavelet decomposition and select a threshold for processing:
  hard threshold method:
    (3) $$Ŵ=\{\begin{array}{c} W,\left|W\right|\geq \lambda \\ 0,\left|W\right|<\lambda \end{array}$$
  soft threshold function:
    (4) $$Ŵ=\{\begin{array}{c} Sign\left(W\right)\left(\left|W\right|-\lambda \right),\left|W\right|\geq \lambda \\ 0,\left|W\right|<\lambda \end{array}$$

where W is the wavelet coefficient and λ is the wavelet threshold.

### 3.2. Limitations of Soft and Hard Threshold Denoising

The key point of wavelet threshold denoising is to choose the appropriate wavelet threshold. The criteria determined by different thresholds correspond to different wavelet threshold denoising methods. The hard threshold and soft threshold are simple and easy to use.

However, hard thresholds and soft thresholds each have disadvantages [18]. The hard threshold method leaves only a large wavelet coefficient, and sets the smaller wavelet coefficient to 0, which causes the signal to oscillate, and the processed wavelet coefficient is discontinuous at the threshold. The soft threshold method instead performs the contraction transformation. Although the continuity is good, there is a constant deviation λ between the processed wavelet coefficients Ŵ and |W|, which affects the degree of approximation of the reconstructed signal and the original signal.

In addition, both the hard threshold method and the soft threshold method set the wavelet coefficient smaller than the threshold to zero, which completely eliminates the useful signal in the noise spectrum, which inevitably leads to a certain deviation between the reconstructed signal and the actual signal.

### 3.3. Improved Threshold Function

A continuity test is performed on the improved threshold function, then the improved wavelet threshold function takes the limit at the threshold λ:

(5) $$\underset{\left|W\right|\to \lambda }{\lim}Ŵ=\{\begin{array}{c} 0,\left|W\right|\geq \lambda \\ \lambda e^{-f},\left|W\right|<\lambda \end{array}$$

When |W| = λ, Ŵ = 0, so the improved threshold function is continuous at |W| = λ, overcoming the disadvantage that the hard threshold function is discontinuous at the threshold.

**Figure 5:**
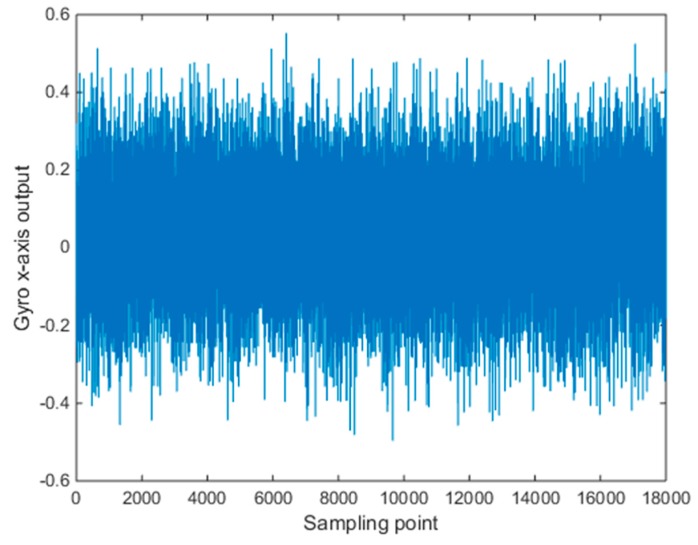
Original signal of X axis gyro.

In order to verify the denoising effect of the improved wavelet threshold algorithm, verify the validity of the improved threshold function, and compare the data of Allan variance analysis before, the data length was 18,000, and shown in Figure 5 are the collected gyro static data.

We used the hard threshold method, the soft threshold method, and the improved wavelet threshold method to filter the same data. The wavelet transform selected the wavelet base db6, the decomposition layer number was five, the threshold value λ = $\sigma \sqrt{2\ln\left(N\right)}$, and σ represents the noise standard deviation. N is the length of the sampled signal. The comparison between the original data and the noise-reduced data is as follows.

**Figure 6:**
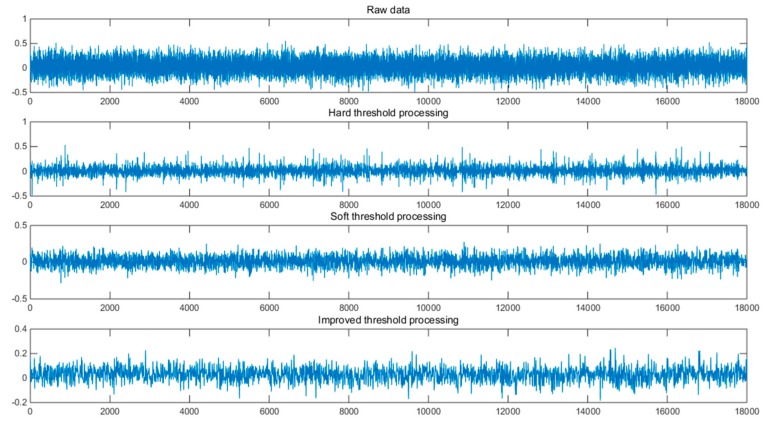
Contrast of denoised data and original data.

It can be seen from Figure 6, above, that after denoising with a hard threshold function, the waveform of the signal will fluctuate somewhat, and the overall observed waveform will be rough. After denoising by soft threshold function, it can be seen that the signal waveform is smoother, but the signal reconstruction accuracy is low, and some useful information may be lost. In the last waveform diagram, the improved wavelet threshold denoising method combined the soft threshold and the hard threshold method to minimize the oscillation of the waveform and suppress the loss of useful information. This improved the reliability of the reconstructed signal.

For a signal with noise, the signal-to-noise ratio of the signal after denoising is larger, and the smaller the mean square error, the better the denoising effect of the signal. As shown in Table 2, the improved threshold denoising method is superior to the traditional hard threshold and soft threshold methods. MSE means square error and SNR is signal to noise ratio.

In addition, the improved threshold function can adjust the degree of shrinkage of the wavelet coefficients according to the size of the wavelet coefficients, which has certain adaptability.

The threshold denoising method as a typical wavelet transform method has the advantages of a simple algorithm and small computational complexity, so it is widely used in signal processing. Applying the threshold denoising method to the noise separation of MEMS gyroscopes is one of the effective ways to improve the signal-to-noise ratio of existing devices.

**Figure 7:**
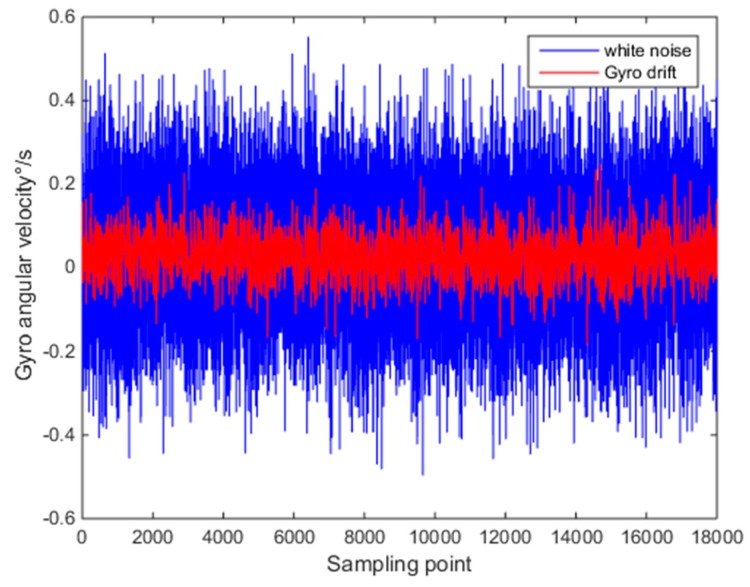
Separated error and white noise.

Then, the improved wavelet threshold method was used to effectively separate the drift error and white noise according to the error separation algorithm of wavelet transform. The drift error and white noise after separation are as shown in Figure 7.

## 4. Determination of the RBF Neural Network Model

In this paper, the RBF neural network proposed by Broomhead and Lowe was used for drift prediction. Compared with other neural networks, the RBF neural network consists of only the input layer, the hidden layer, and the output layer, and is a feedforward local approximation neural network. Therefore, the training speed and convergence speed of the RBF neural network are faster [19].

**Figure 8:**
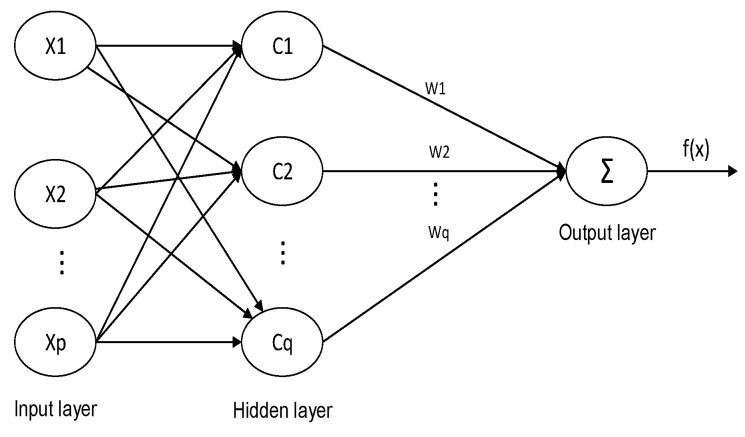
Radial basis function (RBF) neural network structure.

As shown in Figure 8, the input layer node transmits an input signal to the hidden layer; the hidden layer node is formed by a radial function similar to a Gaussian function. The action basis function responds to the local input signal, that is, when the input signal is close to the center of the base function, the hidden layer node produces a larger output, and the output layer node is generally the simplest linear function. It responds to the input mode [20].

Where X = {x1,x2,…xp} is the network input, C = {c1,c2,…cq} is the center of the hidden layer basis function, W = {w1,w2,…wq} is the weight vector, and there is also w0 as the output layer offset. p and q are the dimensions of the network input layer and the number of hidden layer neurons, respectively. This paper chose the Gaussian function as the hidden layer node function:

(6) $$\omega_{i}\left(x\right)=\exp\left(\frac{{\left|\left|x-c_{i}\right|\right|}^{2}}{2\beta_{i}{}^{2}}\right).$$

In the formula, $\omega_{i}$ W is the output of the *i*-th hidden layer node; $c_{i}$ is the center of the *i*-th Gaussian function; the *i*-th Gaussian function scale factor $\beta_{i}$ determines the sensitive domain of the basis function around the center point; the vector norm ‖$x-c_{i}$‖ represents the distance between x and $c_{i}$. General RBF network expressions:

(7) $$f\left(x\right)=\sum_{i=1}^{q}w_{i}\omega_{i}\left(x\right)+w_{0}.$$

It can be seen from Equation (5) that the center and local sensitivity domains of each neuron in the hidden layer of the RBF neural network determine the position and width of the radial basis function. With enough hidden neurons, proper center position, local receptive field and weight, the RBF network can approximate any function with arbitrary precision. The main factor affecting the prediction speed and accuracy is the determination of the input parameters. If the input parameters are inaccurate, it will cause the RBF neural network to generate a large number of neurons in the prediction process, which will cause the prediction speed to decrease, or even lead to non-convergence. The selected sample data cannot estimate the distribution of the entire data. In order to prevent over-fitting, more training data are taken.

**Figure 9:**
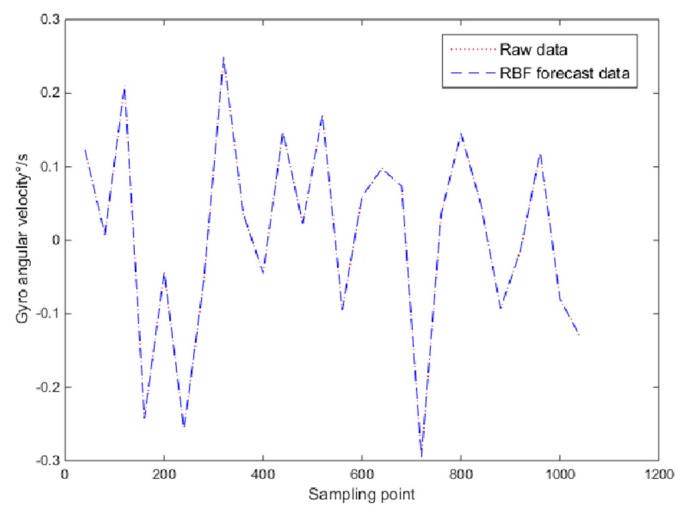
Raw signal and RBF prediction signal.

Therefore, in the prediction estimation, the RBF neural network input layer was set to four and the output layer to one. The first 9.9 × 10^4^ data of the gyro random drift time series were set as the training samples, and the training error accuracy was 5.1 × 10^−6^. The trained neural network predicted the last 1000 data, and the predicted and true values are shown in Figure 9.

**Figure 10:**
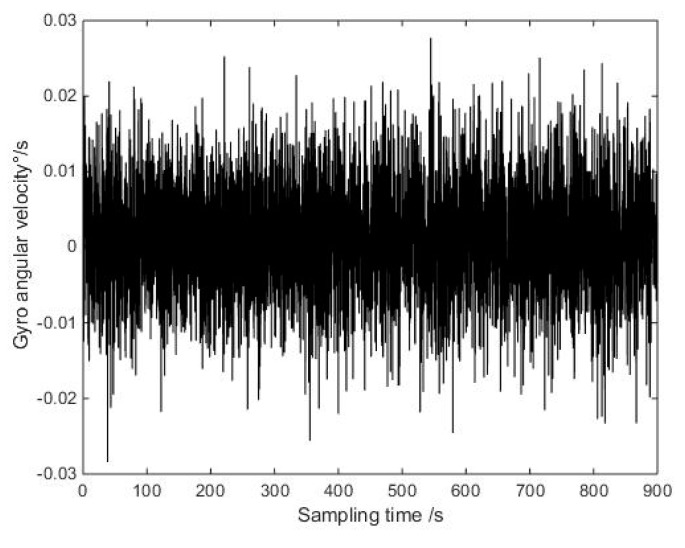
Drift error compensation.

The improved wavelet threshold denoising method was used to separate the drift error from the white noise in the above, and then the RBF neural network was used to model the separated drift error. The compensation effect after modeling is shown in Figure 10. It can be seen that the RBF neural network model established in this paper had good generalization ability and higher precision prediction and compensation ability for MEMS gyro drift error.

**Figure 11:**
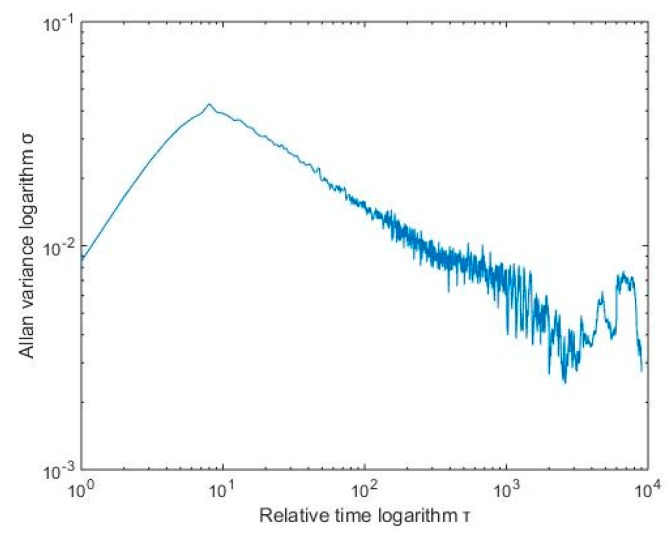
Allan variance curve.

For the RBF neural network modeling compensated signal (X-axis), Allan standard deviation analysis was performed. The results are shown in Figure 11.

The noise figure obtained by Allan data processing is shown in Table 3.

As shown in the above table, after wavelet threshold denoising and RBF neural network modeling compensation, the quantization coefficient was relatively small, indicating that the data acquisition system had high precision, and the angle random walk coefficient and the zero point offset instability coefficient were small, indicating that the detection mode had good stability. The various error coefficients in the Allan analysis of variance were significantly reduced, which indicates that the method can effectively reduce the random error of MEMS gyro performance and improve the accuracy of MEMS gyroscope output information.

## 5. Conclusions

This paper first provided the basic principle of Allan analysis of variance, and analyzed the error coefficients of MEMS gyroscopes, which can play a good role in identification. Then, based on the analysis of conventional wavelet threshold denoising, an improved wavelet threshold denoising method was proposed. This threshold function avoided the complete elimination of the useful signal in the noise and preserved the effective signal as much as possible. Then, based on the analysis of conventional wavelet threshold denoising, an improved wavelet threshold denoising method was proposed. This threshold function avoided the complete elimination of the useful signal in the noise and preserved the effective signal as much as possible. The white noise and drift error of the MEMS gyroscope were successfully separated by the improved wavelet threshold denoising method. The separation of the error terms helped to remove and compensate the error of the MEMS sensor. Finally, the drift error was effectively compensated using the RBF neural network. The experimental results show that in the indoor inertial navigation system, the improved wavelet threshold method was used to decompose the random error and the RBF neural network modeling compensation, which effectively improved the accuracy of the output angle information of the MEMS gyroscope. It is of great significance to improve the accuracy of indoor inertial navigation based on MEMS gyroscopes.

## Figures and Tables

**Table 1:** X-axis values of the parameters Allan variance.

Noise Types	Result
Quantization noise Q/s^−1^	0.09492
Angle random walk N/(°)/$h^{\frac{1}{2}}$	0.04748
Zero bias instability B/(°)/h	0.00670

**Table 2:** X-axis values of the parameters Allan variance.

Threshold Function	SNR	MSE
Hard threshold	34.3	0.049
Soft threshold	38.6	0.023
improvement	44.9	0.0157

**Table 3:** X-axis values of the parameters Allan variance.

Noise Types	Raw Data	RBF Compensation
Quantization noise Q/s^−1^	0.09492	0.04387
Angle random walk N/(°)/$h^{\frac{1}{2}}$	0.04748	0.03292
Zero bias instability B/(°)/h	0.00670	0.00410
